# Phylogenetic Analysis of Lednice *Orthobunyavirus*

**DOI:** 10.3390/microorganisms7100447

**Published:** 2019-10-13

**Authors:** Rebeka Lucijana Berčič, Krisztián Bányai, Daniel Růžek, Enikő Fehér, Marianna Domán, Vlasta Danielová, Tamás Bakonyi, Norbert Nowotny

**Affiliations:** 1 Viral Zoonoses, Emerging and Vector-Borne Infections Group, Institute of Virology, University of Veterinary Medicine Vienna, Veterinaerplatz 1, 1210 Vienna, Austria; rebekalucijana@yahoo.com; 2 Department of Microbiology and Infectious Diseases, University of Veterinary Medicine, Hungária krt. 23-25, 1143 Budapest, Hungary; 3 Institute for Veterinary Medical Research, Centre for Agricultural Research, Hungarian Academy of Sciences, Hungária krt. 21, 1143 Budapest, Hungary; bkrota@hotmail.com (K.B.); feher.eniko@agra.mta.hu (E.F.); doman.marianna@gmail.com (M.D.); 4 Department of Virology, Veterinary Research Institute, Hudcova 296, 621 00 Brno, Czech Republic; ruzekd@paru.cas.cz; 5 Institute of Parasitology, Biology Centre of the Czech Academy of Sciences, Branisovska 31, 370 05 Ceske Budejovice, Czech Republic; 6 National Institute of Public Health, Centre of Epidemiology and Microbiology, Šrobárova 48, 10042 Prague, Czech Republic; vdanielova@seznam.cz; 7 Department of Basic Medical Sciences, College of Medicine, Mohammed Bin Rashid University of Medicine and Health Sciences, Building 14, Dubai Healthcare, Dubai, UAE

**Keywords:** Lednice virus, *Orthobunyavirus*, genome, phylogeny

## Abstract

Lednice virus (LEDV) has been detected in *Culex modestus* mosquitoes in several European countries within the last six decades. In this study, phylogenetic analyses of the complete genome segments confirm that LEDV belongs to the *Turlock orthobunyavirus* (*Orthobunyavirus*, *Peribunyaviridae*) species and is closely related to Umbre, Turlock, and Kedah viruses.

## 1. Introduction

Lednice virus (LEDV) was first isolated from *Culex modestus* mosquitoes in the southeastern part of the Czech Republic (formerly Czechoslovakia) in 1963 [1] and was identified as a bunyavirus [2]. Serological studies revealed its closest antigenic relationship with Turlock, Umbre, and Yaba-1 viruses. Currently LEDV is considered as a separate virus within the species *Turlock orthobunyavirus* (*Orthobunyavirus, Peribunyaviridae*) [3,4]. *C. modestus* is considered as a vector and reservoir of the virus. Antibodies against LEDV were detected in wild birds [5,6] in Czechoslovakia, Austria, Romania, and Germany [5,6,7,8]. The virus can be propagated in the chorioallantoic membrane of chick embryos [9] and in goose, duck, and chick embryo cell cultures [10]. LEDV infection causes viremia and antibody formation in ducklings, goslings, gulls, and coots [11,12]. *Macaca mulatta* monkeys did not develop clinical signs after inoculation with LEDV and virus multiplication was not detected [13]. Extensive research on mosquitoes and potential hosts of LEDV in southern Moravia (Czech Republic) has shown that this virus is tightly linked solely to the littoral zone of large ponds with abundant reed growth [14]. This biotope provides hatching and hiding places as well as food source on nesting water birds for the *C. modestus* vector. *Turlock orthobunyavirus* has been isolated from different *Culex* spp. For example, Turlock virus from *C. tarsalis* [15,16] and *C. erythrothorax* [17] in America; Umbre virus from *C. vishnui* [18], *C. gelidus* [19], and *C. bitaeniorhynchus* [20] in India, and M’Poko virus from *C. cinereus* in Guinea [21]. LEDV was not found in other mosquito species in adjacent inundated areas. Although *C. modestus* occasionally feeds on humans, LEDV has not yet been identified as a human pathogen. Genetic information on LEDV was not available until now. Here we describe the complete genome sequence of three strains of LEDV (strains 110, 6101, and 6118) and infer their genetic relationships with other members of the genus *Orthobunyavirus*.

## 2. Materials and Methods

LEDV strain 110 was isolated in 1963 from *C. modestus* mosquitoes at a fishpond near the village of Lednice, South Moravia, Czechoslovakia [2]. LEDV strains 6101 and 6118 were isolated in 1972 from a pool of 200 *C. modestus* mosquitoes each. The mosquitoes were trapped at Mlynsky pond, Lednice [22]. Low-passage strains were used in this study. LEDV strains were selected for shotgun deep sequencing from cell lysates using the protocols described elsewhere [23]. In brief, after nuclease treatment with a mixture of Turbo DNAse (Ambion, Austin, Texas, United States), RNase I (Thermo Fisher Scientific, Waltham, Massachusetts, United States), and Benzonase (Novagen (Merck), Darmstadt, Germany), the viral RNA was extracted by using the Direct-zol RNA MiniPrep Kit (Zymo Research, Irvine, California, United States). Random primed RT-PCR was carried out using a tagged random hexamer [24] and AMV reverse transcriptase (Promega, Madison, Wisconsin, United States) followed by amplification with DreamTaq DNA polymerase (Thermo Fisher Scientific). Resulting DNA smears extracted from gel slices were used for library preparation compatible with semiconductor sequencing. Sequencing was carried out on Ion Torrent PGM 316 chip using the 200-bp sequencing protocol. Raw sequencing data were evaluated by the CLC Genomics Workbench version 7 (CLC Bio-Qiagen, Aarhus, Denmark). By using a combination of *de novo* assembly and reference sequence mapping, a single consensus sequence was obtained for each of the three viral genomic segments. Sequences of the genome segments were annotated on the basis of positional alignment to the genome sequences of Umbre virus (GenBank accession nos. KP792685–KP792687). Transmembrane domains of glycoprotein precursors were predicted using the same sequences and open-source TMHMM Server v 2.0. (DTU Bioinformatics, Lyngby, Denmark). Multiple alignments of the nucleotide sequences were generated by using the ClustalW algorithm. The evolutionary histories of the three genome segments were inferred by using the maximum likelihood method and general time-reversible model [25]. Trees with the highest log likelihood are shown. The percentages of trees in which the associated taxa clustered together are shown next to the branches. Initial trees for the heuristic search were obtained automatically by applying Neighbor-Join and BioNJ algorithms to matrices of pairwise distances estimated using the maximum composite likelihood (MCL) approach, followed by selection of topology with superior log likelihood values. Discrete gamma distributions were used to model evolutionary rate differences among sites (5 categories (+G)). Trees are drawn to scale, with branch lengths measured in the number of substitutions per site. Evolutionary analyses were conducted in MEGA X [26].

The complete genome sequences of the three genome segments of three LEDV strains were identified, annotated, and deposited in GenBank database under accession numbers MK514119–MK514127.

## 3. Results

Sequence analysis indicated a classical *Orthobunyavirus* genome organization of the LEDV strains. The small (S) segments of all three strains contain 1010 nucleotides. The ORF starting at nucleotide (nt) 122 codes for a 236-amino acid (aa) long nucleocapsid (N) protein. The S segment sequences of strains 6101 and 6118 are identical and differ in 9 nt from strain 110. The nucleocapsid protein differs in one aa (valine_22_alanine) in strain 110 compared with 6101 and 6118. Sequences of strains 6101 and 6118 contain another ORF starting at nt 159, coding for the small non-structural (NSs) protein. In strain 110, the transition C_186_T introduced a premature stop codon TAG truncating the NSs protein to 9 aa.

The medium (M) segments contain 4498 (strain 110) and 4497 (strains 6101 and 6118) nt, respectively. The ORF starting at nt 33 codes for a 1461 aa long Gn-NSm-Gc glycoprotein precursor polyprotein. The M segment sequence of strain 110 differs in 54 nt from strains 6101 and 6118, and these two strains differ in six nt from each other. The polyprotein sequence of strain 110 differs in 24 aa from 6101 and 6118, while these two strains differ in six aa from each other.

**Figure 1:**
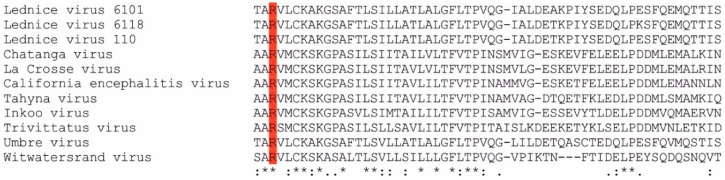
Alignment of selected *Orthobunyavirus* M segment amino acid sequences. The conserved arginine residue (signal peptidase cleavage motif between Gn and NSm proteins) is highlighted with red background. Symbols in the bottom line denote conserved sequence (*), conservative mutations (:), semi-conservative mutations (.), and non-conservative mutations ( ).

Sequence analysis of the M segment predicted a conserved arginine residue as a signal peptidase cleavage motif located between Gn and NSm proteins of orthobunyaviruses [27]. This residue was identified in all LEDV strains at aa position 303 (Figure 1).

Signalase cleavage sites between NSm and Gc could not be predicted in the LEDV strains by using the algorithms recommended by Briese et al. [28]; however, a conserved cysteine residue was found in all the investigated orthobunyaviruses after the cleavage site identified in Bunyamwera virus [27]. Six transmembrane domains of the glycoprotein precursors were predicted between aa positions 205–224, 229–248, 313–335, 364–386, 455–477, and 1396–1418 in strain 110 and 205–224, 209–248, 330–335, 364–386, 455–477, and 1396–1418 in strains 6101 and 6118. Putative N-glycosylation sites of the glycoprotein precursor were predicted in all LEDV strains at aa positions 37, 61, 594, 663, 703, 905, and 1175. An additional putative N-glycosylation site was found in strains 6101 and 6118 at aa position 601.

The large (L) segments contain 6946 nt, the ORF starting at nt 45 codes for a 2245 aa RNA-dependent RNA polymerase (RdRp). The L-segment sequences of strain 110 differ in 126 and 127 nt from strains 6101 and 6118, respectively; and the latter two strains differ in three nt from each other. The RdRp sequence of strain 110 differs in six and eight aa from 6101 and 6118, respectively; while these two strains differ in two aa from each other.

The N-termini of the bunyavirus L proteins contain regions 1 and 2. In each region, stretches of conserved residues are centered on the strictly conserved dipeptide (dipeptide PD for region 1 and RY for region 2). The third region contains six conserved blocks (also conserved between the L proteins of most bunyaviruses): Premotif A (located upstream from motif A, containing three strictly conserved basic residues; the distances between them are also preserved), motifs A, B, C, and D (defining the so-called polymerase module, which are present in all RNA-dependent polymerases analyzed so far), and additional motif E (containing the tetrapeptide E(F/Y)VS, located downstream of motif D, conserved in segmented negative-stranded RNA virus polymerase) [29]. As all bunyavirus RNA polymerases, LEDV also contains a conserved N-terminal, influenza-like endonuclease domain, essential for viral cap-dependent transcription [30].

**Figure 2:**
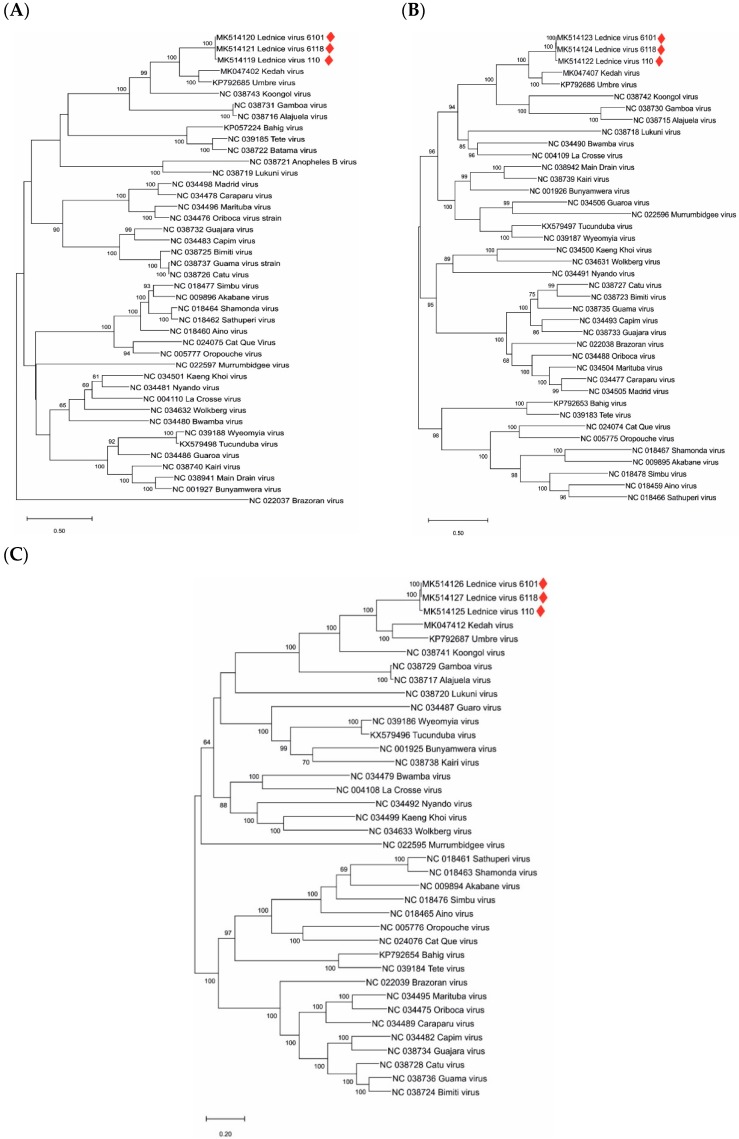
Phylogenetic analysis of orthobunyavirus complete genome sequences. (**A**): S segment; (**B**): M segment, (**C**): L segment. Sequences are labeled by codes containing the GenBank accession number and virus name. LEDV sequences are marked with red lozenges. The phylograms were generated with the maximum likelihood statistical method; bootstrap percentage values of 500 replicates above 60% are displayed. Horizontal bars on the left represent genetic distances.

The genome segment sequences of the LEDV strains were aligned with all complete genome sequences of orthobunyaviruses available in public databases. The highest identity rates were found with Umbre virus (60% nt and 73% aa identity in segment S, 71% nt and 76%–77% aa identity in segment M, and 73% nt and 83% aa identity in segment L). Phylograms indicating inferred genetic relationships between LEDV and other orthobunyaviruses are shown in Figure 2A–C.

## 4. Discussion

Our study provides the first genetic evidence that LEDV virus is closely related to Umbre virus, and hence, it is a variant of *Turlock orthobunyavirus*. However, according to the ICTV species demarcation criteria, aa sequences of the N proteins of species within the *Orthobunyavirus* genus which differ by more than 10% are considered distinct virus species (ICTV 9th Report), particularly, if differences in geographic distributions are also taken into consideration [31]. This difference between Lednice and Umbre viruses amounts to 27%, and Turlock virus was so far only detected in North America, Umbre virus in India, and Lednice virus in Europe [20,32].

Recently, a novel *Orthobunyavirus*, named the Kedah fatal kidney syndrome virus (KFKSV), was reported as the etiologic agent of severe kidney disease in broiler chickens in Malaysia [33]. KFKSV is genetically closely related to Umbre virus and the first representative of *Turlock orthobunyavirus* with significant pathogenicity. The genetic distance between LEDV and KFKSV is similar to those between LEDV and Umbre virus (Figure 2). In medical and veterinary diagnostic virology laboratories, samples of tubulonephrosis and interstitial nephritis cases are not tested routinely for *Orthobunyavirus* infections. Therefore, targeted studies investigating the role of *Turlock orthobunyavirus*, including LEDV, in kidney diseases of animals and humans are suggested. This study provides genetic information for the accurate identification of these orthobunyaviruses.
